# Thiol/disulfide homeostasis impaired in patients with primary Sjögren's syndrome

**DOI:** 10.5937/jomb0-27281

**Published:** 2021-06-05

**Authors:** Cakmak Nuray Yilmaz, Emin Gemcioglu, Salih Baser, Sükran Erten, Ozcan Erel

**Affiliations:** 1 Ankara City Hospital, Department of Internal Medicine, Ankara, Turkey; 2 Yıldırım Beyazıt University, Ankara City Hospital, Department of Internal Medicine, Ankara, Turkey; 3 Yıldırım Beyazıt University, Ankara City Hospital, Department of Internal Medicine, Division of Rheumatology, Ankara, Turkey; 4 Yıldırım Beyazıt University, Ankara City Hospital, Department of Biochemistry, Ankara, Turkey

**Keywords:** primary Sjögren's syndrome, oxidative stress, thiol/disulphide homeostasis, primarni Sjögrenov sindrom, oksidativni stres, tiol-disulfidna homeostaza

## Abstract

**Background:**

Primary Sjögren's syndrome (pSS) is a disease associated with the overexpression of proinflammatory cytokines, and oxidative stress is one of the factors responsible for its etiopathogenesis. This study aimed to investigate the thiol/disulphide homeostasis in pSS patients.

**Methods:**

The study included 68 pSS patients and 69 healthy controls. Thiol/disulphide homeostasis (total thiol, native thiol, and disulphide levels) was measured using the automatic spectrophotometric method developed by Erel and Neselioglu, and the results of the 2 groups were compared.

**Results:**

The gender and age distributions of the pSS and control groups were similar (*P* = 0.988 and *P* = 0.065). Total thiol and native thiol levels were lower in the pSS group than in the control group (470.08 ± 33.65 µmol/L vs. 528.21 ± 44.99 µmol/L, *P* < 0.001, and 439.14 ± 30.67 µmol/L vs. 497.56 ± 46.70 µmol/L, *P* < 0.001, respectively). There were no differences in disulphide levels between groups [17.00 (range 0.70-217.0) µmol/L vs. 14.95 (range 2.10-40.10) µmol/L, *P* = 0.195].

**Conclusions:**

It was concluded that the thiol/disulphide balance shifted towards disulphide in patients with pSS.

## Abstract (sr)

**Uvod:**

Primarni Sjögrenov sindrom (pSS) je bolest povezana sa prekomernom ekspresijom proinflamatornih citokina, dok je oksidativni stres jedan od faktora odgovornih za njegovu etiopatogenezu. Cilj ove studije je bio da ispita tioldisulfidnu homeostazu kod pacijenata sa pSS-om.

**Metode:**

Studija je obuhvatila 68 pacijenata sa pSS-om i 69 zdravih kontrolnih pojedinaca. Homeostaza tiol-disulfida (nivoi ukupnog tiola, nativnog tiola i disulfida) je izmerena pomoću automatske spektrofotometrijske metode koju su razvili Erel i Neselioglu. Rezultati dve grupe su upoređeni.

**Rezultati:**

Raspodela po polu i starosti pacijenata sa pSSom i pojedinaca u kontrolnoj grupi je bila slična (*P* = 0,988 i *P* = 0,065). Ukupni nivoi tiola i nativnog tiola bili su niži u grupi sa pSS-om nego u kontrolnoj grupi (470,08 ± 33,65 µmol/L naspram 528,21 ± 44,99 µmol/L, *P* <0,001 i 439,14 ± 30,67 µmol/L naspram 497,56 ± 46,70 µmol/L, *P* <0,001, respektivno). Nije bilo razlika u nivoima disulfida između grupa [17,00 (opseg 0,70-217,0) µmol/L naspram 14,95 (opseg 2,10-40,10) µmol/L, *P* = 0,195].

**Zaključak:**

Zaključeno je da se ravnoteža tiol/disulfid pomera prema disulfidu kod pacijenata sa pSS-om.

## Introduction

Sjögren's syndrome (SS) is a systemic chronic inflammatory disease characterized by the lymphocytic infiltration of exocrine organs [1] [2]. It is further divided into 2, as primary and secondary SS, depending on the other accompanying autoimmune diseases. Primary SS (pSS) is an autoimmune disease characterized by lymphocytic infiltration and destruction of the salivary and tear glands, and systemic auto antibody production against SS-A/Ro and SS-B/La ribonucleoprotein particles. Although etio pathogenesis is not clearly understood, the current theory is that environmental factors trigger autoimmunity in genetically predisposed individuals. SS is associated with the overexpression of proinflammatory cytokines, including tumour necrosis factor-alpha, interleukin (IL)-7, IL-1 beta, IL-6, IL-10, IL-17, IL-18, and interferon-gamma [3]. Together with the increased inflam matory processes, the increased reactive oxygen radicals are also thought to increase the formation of immune complexes and exacerbate the destruction of exocrine glands. It has been demonstrated that the oxidative stress associated with accompanying autoimmune diseases (such as systemic lupus erythematosus, rheumatoid arthritis (RA), and systemic sclerosis) takes part in the etiopathogenesis of SS. Therefore, it is plausible that oxidative stress plays a role in the etiopathogenesis of SS.

Oxidative damage in the body is prevented by enzymatic or non-enzymatic antioxidant mechanisms [4]. Thiols are a principal member of both the enzymatic and non-enzymatic intracellular antioxidant systems. Thiols are also known as mercaptans. They are organic compounds that are formed by binding sulphur and hydrogen atoms to carbon atoms [5]. The thiol pool in the plasma consists mainly of thiols, such as albumin, proteins, low molecular weight cysteine, cysteine-glycine, glutathione, homocysteine, and gamma-glutamylcysteine [6]. When the level of free oxygen radicals' increases (i.e., increased oxidative stress), the plasma thiol/disulphide balance shifts against thiol, and disulphide levels increase [7] [8].

To the best of our knowledge, there are no studies that have investigated oxidative status via the measurement of native thiol, total thiol, and disulphide levels in patients with pSS. Therefore, it was aimed herein to evaluate the oxidative status via these parameters in patients with pSS.

## Materials and Methods

### Study Population

Sixty-eight patients with pSS and 69 healthy individuals were included in the study. Patients with cardiovascular diseases, cerebrovascular diseases, kidney failure, liver failure, infections, malignancies, and individuals that used antioxidants, multivitamins, cigarettes, or alcohol were excluded from the study. SS was diagnosed according to the criteria defined by the 2016 American College of Rheumatology/European League Against Rheumatism [9].

The demographic and clinical characteristics (age, sex, xerostomia or xerophthalmia that had been present for 3 months, presence of arthritis, myositis, or vasculitis, pulmonary, renal, hematological, or hepatological involvement, presence of Raynaud's phenomenon, and Schirmer tests and salivary gland biopsy results) and laboratory results [hemogram, aspartate transaminase (AST), alanine transaminase (ALT), glucose, urea, creatinine, erythrocyte sedimentation rate (ESR), C-reactive protein (CRP), anti-Ro antibody (anti-SSA), anti-La antibody (anti-SSB), antinuclear antibody (ANA), rheumatoid factor (RF), total thiols (-SH + -S-S-), native thiols (-SH) and dynamic disulphide (-S-S-)] of the patients were recorded. The study was approved by the ethical board of the institution, and informed consent was obtained from all participants (Approval date and number:18.09.2019/26379996/96).

### Biochemical Parameters

Biochemical parameters (AST, ALT, glucose, urea, and creatinine) were measured using standard laboratory methods (Cobas 501; Roche Diagnostics, Germany). The hemogram was evaluated on Sysmex XN-1000 (Sysmex Corporation, Kobe, Japan) analyzer, and serological parameters were studied using the nephelometric method (Siemens BN-11).

### Thiol/Disulphide Homeostasis

For the assessment of the thiol/disulphide homeostasis, blood samples were centrifuged at 1500 rpm for 10 min and stored at -80°C until assessment. After all of the samples had been collected, they were simultaneously evaluated for oxidative stress parameters by the same laboratory technician using the same device. Native thiol, total thiol, and disulphide levels were assessed using the novel and fully automated assay developed by Erel and Neselioglu, which is based on the reduction of dynamic disulphide bonds to free thiol groups by sodium borohydride (NaBH4) [10]. Formaldehyde was added to eliminate excess NaBH4 to avoid the extra reduction of DTNB (5,50-dithiobis-[2-nitrobenzoic acid]), and further reduction of the formed disulphide bonds after the DTNB reaction. Ellman's reagent was used to measure the total thiol content.

Next, the native thiol content was subtracted from the total thiol content, and 50% of this difference revealed the quantity of the disulphide bond. An automated clinical chemistry analyser (Cobas 501) was used to measure the amount of native thiol and disulphide. The serum thiol and disulphide values were presented as µmol/L.

### Statistical Analysis

All statistical analyses were performed with IBM SPSS Statistics 21.0 (Armonk, NY, USA) software. The Shapiro-Wilk test was used to evaluate compliance with the normal distribution. Descriptive analyses were presented as the mean ± standard deviation (SD) for normally distributed variables and median and range (min-max) for non-normally distributed variables. Categorical variables were presented as numbers and percentages. The 2 groups were compared using the Student t-test and 1-way ANOVA for parametric variables, and the Kruskal-Wallis test for non-parametric variables. *P* < 0.05 was considered statistically significant.

## Results

Of the pSS patients, 66 were female, and 2 were male, and of the controls, 67 were female, and 2 were male (*P *= 0.988). The mean ages of the patient and control groups were 53.71±8.17 and 51.04±8.57 years, respectively (P = 0.065). Of the pSS patients, 64.7% had Raynaud's phenomenon, 57.4% were anti-SSA-positive, and 69.1% were anti-SSB-positive. The Schirmer's test result was 2 mm in 30.9% of the patients. According to the Chisholm-Mason classification [11] [12], the salivary gland biopsy results of the pSS patients were as follows: stage 1 in 5.9%, stage 2 in 22.1%, stage 3 in 38.2%, and stage 4 in 33.8%. Of the pSS patients, 58.8% had xerostomia, and 52.9% had xerophthalmia. Data regarding the symptoms and organ involvement of the pSS patients are presented in Table 1.

**Table 1:** Clinical findings and autoimmune parameters of the patients with pSS ANA, antinuclear antibody; Anti-SSA, anti-SS-related antigen A; Anti-SSB, SS-related antigen B, RF, rheumatoid factor.

Variables		n	%
Xerostomia	No	28	41.2
	Yes	40	58.8
Xerophthalmia	No	32	47.1
	Yes	36	52.9
Arthritis	No	48	70.6
	Yes	20	29.4
Pulmonary involvement	No	35	51.5
	Yes	33	49.5
Renal involvement	No	62	91.2
	Yes	6	8.8
Hematological involvement	No	34	50
	Yes	34	50
Raynaud’s phenomenon	No	36	52.9
	Yes	32	47.1
Hepatic involvement	No	65	95.6
	Yes	3	4.4
Myositis	No	66	97.1
	Yes	2	2.9
Vasculitis	No	65	95.6
	Yes	3	4.4
Minor salivary gland biopsy	Stage 1	4	5.9
	Stage 2	15	22.1
	Stage 3	26	38.2
	Stage 4	23	33.8
Schirmer’s test	≤2 mm	21	30.9
	2–5 mm	0	0
	≥5 mm	47	69.1
ANA	Negative	34	50
	+	10	14.7
	++	4	5.9
	+++	20	29.4
Anti-SSA	Negative	29	42.6
	Positive	39	57.4
Anti-SSB	Negative	21	30.9
	Positive	47	69.1
RF	Negative	24	35.3
	Positive	44	64.7

The ESR, CRP, and AST levels of the patient group were significantly higher and the leukocyte values were lower than in the control group (*P* < 0.001, *P* < 0.001, *P* < 0.001, and *P*= 0.009, respectively). The total thiol and native thiol levels were statistically lower in the pSS patients when compared to the controls (470.08±33.65 µmol/L vs. 528.21±44.99 µmol/L, *P* < 0.001, and 439.14±30.67 µmol/L vs. 497.56±46.70 µmol/L, *P* < 0.001, respectively). Although the disulphide levels were higher in the pSS group when compared to the control group, this difference was not statistically significant [17.00 (range 0.70-217.0) µmol/L vs. 14.95 (range 2.10-40.10) µmol/L, *P *= 0.195] (Table 2).

**Table 2:** Comparison of thiol/disulfide levels, demographic and laboratory findings of the pSS and control groups Note: Parameters are expressed as the means ± SD and medians [interquartile range]. P < 0.05 was considered statistically significant. AST, Aspartate transaminase; ALT, alanine transaminase; ESR, erythrocyte sedimentation rate, CRP, C-reactive protein.

	Control (n = 69)	pSS (n = 68)	P-value
Age (years)	51.04 ± 8.57	53.71 ± 8.17	0.065
Gender (female/male)	67/2	66/2	1.000
Hemoglobin (g/L)	137.2 ± 13.7	131.5 ± 12.3	0.015
Leukocyte (10^9^/L)	7.12 (4.26–13.42)	6.42 (3.17–15.67)	0.009
Platelet count (10^9^/L)	265.25 ± 63.49	253.61 ± 62.32	0.294
Glucose (mmol/L)	4.83 (4.11–6.72)	5 (4.05–6.83)	0.506
Urea (mmol/L)	7.96 (4.39–16.53)	9.21 (1.18–31.92)	0.063
Creatinine (mmol/L)	58.34 (38.01–97.24)	54.81 (41.55–97.24)	0.019
AST (U/L)	15.00 (10.00–46.00)	19.00 (8.00–42.00)	<0.001
ALT (U/L)	15.00 (8.00–35.00)	17.00 (7.00–53.00)	0.224
ESR (mm/h)	13.00 (1.00–30.00)	24.00 (1.00–59.00)	<0.001
CRP (g/L)	3.27 (3.13–31.00)	3.48 (3.20–99.00)	<0.001
Total thiol (µmol/L)	528.21 ± 44.99	470.08 ± 33.65	<0.001
Native thiol (µmol/L)	497.56 ± 46.70	439.14 ± 30.67	<0.001
Disulfide (µmol/L)	14.95 (2.10–40.10)	17.00 (0.70–217.0)	0.195

The total thiol, native thiol, and disulphide levels were similar in patients with and without xerostomia or xerophthalmia (*P* > 0.05 for all of the parameters). The total thiol, native thiol, and disulphide levels were not significantly associated with the Schirmer's test or minor salivary gland biopsy results (*P* > 0.05 for all of the parameters). Moreover, the thiol/disulphide parameters of the anti-SSA- and anti-SSB-positive patients were similar to those of the anti-SSA- and anti-SSB-negative patients (*P* > 0.05 for all of the parameters) (Table 3).

**Table 3:** Comparison of thiol/disulfide levels of the pSS patients according to xerostomia, xerophthalmia, Schirmer’s test, minor salivary gland biopsy, and some laboratory results Anti-SSA, anti-SS-related antigen A; Anti-SSB, anti-SS-related antigen B.

		Total thiol (µmol/L)	P-value	Native thiol (µmol/L)	P-value	Disulfide (µmol/L)	P-value
Xerostomia	Yes (n = 40)	466.74 ± 35.05	0.341	437.77 ± 30.97	0.631	15.35 (0.70–217)	0.340
	No (n = 28)	474.83 ± 33.05		441.49 ± 31.20		17.70 (4.40–74)	
Xerophthalmia	Yes (n = 36)	470.82 ± 33.10	0.849	440.82 ± 30.18	0.682	17.65 (0.70–217)	0.970
	No (n = 32)	469.22 ± 35.97		437.69 ± 32.04		16.45 (3.05–74)	
Schirmer’s test	≤2 mm (n = 21)	459.54 ± 36.77	0.090	433.26 ± 33.41	0.298	15.9 (0.70–74)	0.239
	≥5 mm (n = 47)	474.77 ± 32.33		441.91 ± 29.75		17.0 (4.4–217)	
Minor salivary gland biopsy	Stage 1 (n = 4)	488.70 ± 21.31	0.348	441.70 ± 15.89	0.221	22.37 (16.55–32.70)	0.309
	Stage 2 (n = 15)	461.90 ± 26.57		435.58 ± 19.68		13.52 (3.10–74)	
	Stage 3 (n = 26)	475.15 ± 34.71		442.88 ± 34.24		17.7 (3.05–27.60)	
	Stage 4 (n = 23)	468.18 ± 38.38		437.17 ± 35.17		17.0 (0.70–217)	
Anti-SSA	Negative (n = 29)	476.50 ± 36.09	0.184	443.84 ± 30.71	0.299	17.8 (0.70–74)	0.324
	Positive (n = 39)	465.29 ± 32.43		435.88 ± 30.98		13.97 (3.05–217)	
Anti-SSB	Negative (n = 21)	479.84 ± 32.92	0.116	447.54 ± 24.51	0.143	18.1 (3.05–74)	0.421
	Positive (n = 47)	465.70 ± 34.24		435.57 ± 32.96		14.75 (0.70–217)	

## Discussion

Although oxidative stress is involved in the pathophysiology of several chronic conditions, there have been a limited number of studies evaluating the relationship between SS and oxidative stress. To the best of our knowledge, the current study was the first to investigate thiol/disulphide homeostasis in pSS. In this study, it was observed that the total thiol and native thiol levels were reduced in the pSS patients when compared to the healthy controls, there was a statistically insignificant increase in the disulphide levels, and the dynamic thiol/disulphide balance shifted towards disulphide.

There is an association between proinflammatory states and oxidative stress. Moreover, studies have suggested that oxidative stress is involved in the pathogenesis of SS. A study by Kurimoto et al. [13] found that the antioxidant thioredoxin had increased in the salivary gland biopsies of SS patients when compared to the controls, and they evaluated this as a protective mechanism against oxidative stress in SS. Cejková et al. [14] measured antioxidant markers, such as superoxide dismutase, catalase, and glutathione peroxidase, in the conjunctival epithelium and showed that their levels decreased with increasing xerophthalmia. Furthermore, oxidative stress markers were found to have increased in plasma and lip biopsy samples of SS patients [15] [16].

Thiols are crucial organic compounds that contain sulfhydryl groups, which play an important role in the oxidant/antioxidant mechanism [17]. The thiol/disulphide balance is crucial to the organism. ROS increase when the balance shifts towards disulphide [18]. Native thiols are molecules containing nonreduced functional thiol groups. As a part of the antioxidant system, native thiols decrease when oxidative stress increases. Total thiol levels reflect the sum of both oxidized and non-oxidized thiols. Plasma thiol and disulphide levels can be measured both separately and combined with the novel assay developed by Erel and Neselioglu in 2014 [10].

The thiol/disulphide balance is impaired in many diseases, such as myocardial infarction, preeclampsia, polycystic kidney disease, diabetes mellitus (DM), and cancer. Disulphide levels increase in degenerative diseases, such as DM, obesity, pneumonia, and familial Mediterranean fever, and the balance shifts towards thiol in proliferative diseases, such as multiple myeloma, bladder cancer, colon cancer, and kidney cancer [10]. A recent study found that native thiol levels were high and disulphide levels were low in patients with fibromyalgia when compared to the healthy controls, and it was reported that in fibromyalgia, the thiol/disulphide balance was similar to that in benign proliferative diseases and that this was due to the proliferative pattern rather than inflammation [19]. Our hypothesis is also supported by the results of some studies that include autoimmune and inflammatory diseases. Dealing with examples of autoimmune diseases, one study compared patients with RA and healthy controls and observed that the thiol/disulphide balance shifted towards disulphide in the RA patients [20]. According to another study which compared the Graves' patients, the ratios of native thiols, total thiols, and native thiols/total thiols were lower; and the ratios of disulphide/native thiol and disulphide/total thiol were higher in the patient group comprehensively to the healthy controls [21]. The results of a study that included Celiac patients showed that total and native thiol levels in celiac patients were lower while disulphide level, disulphide/total thiol, and disulphide/native thiol ratios were higher compared to the healthy people; and also dynamic equilibrium was found shifted to disulphide side [22]. Another study that contained autoimmune gastritis patients showed altered thiol/disulphide homeostasis compared to the healthy group, and the dynamic balance was shifted through disulphide form [23]. On the other hand, to present examples of inflammatory diseases, some studies compared Psoriasis patients and healthy controls; and according to results in the patient group, disulphide, disulphide/native thiol, and disulphide/total thiol were significantly higher, but native thiol and native thiol/total thiol were significantly lower compared to healthy controls. This showed that psoriasis patients had a higher increase in oxidants compared to the healthy group [24] [25]. In a study with pregnant women with Familial Mediterranean Fever, disulphide levels were higher in patients compared to controls [26]. In another study with FMF patients, the relation of thiol/disulphide imbalance and colchicine resistance was evaluated. According to the results, native thiol and total thiol levels were lower in colchicine resistance cases compared to those not colchicine resistance ones. Also, disulphide levels were higher in colchicine resistance cases [27].

In the current study, the total thiol and native thiol levels in the pSS patients were significantly lower than in the control group, as was the case in patients with RA. This suggested that antioxidation had decreased in patients with pSS, indirectly increasing oxidation. The fact that disulphide levels did not increase as expected, despite the fact that native thiols decreased, may have been because these molecules could not be measured due to being converted into advanced oxidation products. It should be noted that the subjects herein with pSS were on a variety of drugs, including immunosuppressives and corticosteroids. Methotrexate is known to reduce oxidative stress by reducing free radicals, such as superoxide (O^-^_2_), and reactive oxygen particles [28]. The reason that the disulphide levels did not increase in the patients herein may have been because of the antioxidation effects of the medications used by the patients, such as methotrexate. It is not known precisely how the drug combinations used by pSS patients affect the thiol/disulphide balance. Besides, the reason why the thiol/disulphide balance did not change with anti-SSA or anti-SSB positivity, salivary gland biopsy results, xerostomia, or xerophthalmia may have been ascribed to medication use as well.

This study had several limitations. First, whether the patients and controls had consumed thiol-containing nutrients was investigated only through anamnesis. Second, the pSS patients were under medical treatment, and the effects of the drugs that they used on the oxidative parameters were not evaluated. Finally, the relationship between disease duration and the oxidative parameters was not evaluated in this study.

In conclusion, the total thiol and native thiol levels decreased in patients with pSS, suggesting that antioxidation was impaired in these patients, and the oxidation/antioxidation mechanism may play a role in the pathogenesis of the disease. To fully evaluate how the thiol/disulphide mechanism is altered in pSS, prospective studies with larger samples that include recently diagnosed pSS patients, who have not yet started treatment, are needed.

## Funding

The author(s) received no financial support for the research, authorship, and/or publication of this article.

## Conflict of interest statement

All the authors declare that they have no conflict of interest in this work.
